# High male fertility in males of a subdioecious shrub in hand-pollinated crosses

**DOI:** 10.1093/aobpla/plw067

**Published:** 2016-10-26

**Authors:** Hui Wang, Michinari Matsushita, Nobuhiro Tomaru, Michiko Nakagawa

**Affiliations:** 1 Graduate School of Bioagricultural Sciences, Nagoya University, Chikusa-ku, Nagoya 464-8601, Japan; 2 School of Life Science, Shandong University, Jinan, China; 3 Forest Tree Breeding Center, Forestry and Forest Products Research Institute, Hitachi, Japan

**Keywords:** Dioecy, *Eurya japonica*, fruit set, male reproductive success, siring success, subdioecy

## Abstract

Female reproductive success in females versus hermaphrodites has been well documented. However, documenting a potential advantage in male fertility of male versus hermaphrodite individuals in subdioecious species is also essential for understanding the evolutionary pathway toward dioecy from hermaphroditism via gynodioecy. Siring success in terms of fruit set, fruit mass, number of seeds and mean seed mass was compared by hand-pollinated crosses in the subdioecious shrub *Eurya japonica*. The pollen was from male and hermaphrodite individuals, and the pollen recipients were females and hermaphrodites. Seed quality was also evaluated in terms of seed germination rate, seed germination day and seedling survival. Overall, pollen from males sired more fruits of larger size and more seeds than did pollen from hermaphrodites. The male advantage was observed when pollen recipients were females, whereas no effect was found in hermaphrodite recipients. Pollen from males also produced better quality seeds with higher germination rate and sooner germination day. Although these results could also be explained by a higher pollen load for crosses with male pollen donors, we took care to saturate the stigma regardless of the pollen donor. Therefore, these results suggest that male individuals of *E. japonica* have advantages in male fertility in terms of both quantity and quality. Our previous studies indicated that females exhibit higher female reproductive success compared with hermaphrodites. Thus, both the female and male functions of hermaphrodites are outperformed by females and males, respectively, raising the possibility that the subdioecious *E. japonica* at this study site is entering the transitional phase to dioecy along the gynodioecy–dioecy pathway.

## Abstract (precis)

Male individuals exhibited an advantage in male fertility in terms of both quantity and quality compared with hermaphrodites in hand-pollinated crosses in the subdioecious species Eurya japonica. This male advantage was prominent when the mother trees were female individuals rather than hermaphrodites. Given that the reproductive success of females is higher than that of hermaphrodites and that hermaphrodites are self-incompatible in E. japonica, pollen limitation may inhibit the shift and permit the persistence of hermaphrodites in this E. japonica population. The subdioecious study population may be entering a transition stage to dioecy along the gynodioecy-dioecy pathway.

## Introduction

Plants have diverse and complex sexual expression, and mixtures of individuals bearing various combinations of female, male and hermaphrodite flowers are sometimes observed within a single population. Although the majority of flowering plants are hermaphrodites, dioecy has arisen repeatedly and is found in 5–6 % of species across 43 % of angiosperm families (Renner and Ricklefs 1995; Heilbuth 2000; Charlesworth 2002; Barrett and Hough 2013; Renner 2014). The gynodioecy–dioecy pathway is considered one of the most important evolutionary routes from hermaphroditism to dioecy via gynodioecy. In this pathway, female individuals (male-steriles) invade a hermaphrodite population, then, hermaphrodites are replaced by males. However, little is known about the differences in male fertility between males and hermaphrodites, despite a great deal of empirical evidence regarding higher female fertility compared with hermaphrodites in the initial stages of the gynodioecy–dioecy pathway (Spigler and Ashman 2012). In subdioecious species, female, male and hermaphrodite individuals coexist within a population, and this sexual system is thought to occupy a later transitional position along the evolutionary pathway from hermaphroditism to dioecy via gynodioecy (Darwin 1877; Charlesworth and Charlesworth 1978). To successfully maintain male individuals in a subdioecious population, males are required to have greater male fertility compared with hermaphrodites, although this hypothesis has rarely been examined (Charlesworth and Charlesworth, 1978; McCauley and Brock 1998; Ashman 2006; Spigler and Ashman 2012). Therefore, clarification of male fertility in subdioecious species will contribute to our understanding of the transition at the later stages of the gynodioecy–dioecy pathway.

In several subdioecious species, males have been reported to exhibit greater male fertility than hermaphrodites in terms of pollen or flower production and pollen viability (Aguirre *et al.* 2007; Philipp *et al.* 2009). However, there have been few comparative studies of siring success (a direct determinant of male fertility) of males and hermaphrodites. Hand-pollination treatments using mixed pollen from a male and a hermaphrodite individual followed by paternity assessment using genetic analyses have documented much higher siring success by males compared with hermaphrodites in subdioecious *Fraxinus excelsior* (Morand-Prieyr *et al.* 2003), but approximately similar siring success in subdioecious *Silene acaulis* (Philipp *et al.* 2009). Moreover, male fertility is markedly affected not only by the quantity of fruit and seed production (siring success) but also by progeny quality, i.e. seed germination rate and seedling survivorship. To date, no studies have examined differences in the fitness of progeny sired by males versus hermaphrodites, which is essential to fully understand the male fertility of males versus hermaphrodites.

The compatibility between pollen donor and pollen recipient may also influence differences in male fertility between males and hermaphrodites. Previous studies have used either only hermaphrodites (Morand-Prieyr *et al*. 2003) or only females (Philipp *et al.* 2009) as pollen recipients in hand-pollinated crosses. When females differ from hermaphrodites in their receptivity to pollen from males and hermaphrodites, the pollen recipients could affect siring success. That is, the differences in male fitness may occur only when females or hermaphrodites are the pollen receivers. Thus, studies should use both female and hermaphrodite individuals as pollen recipients to accurately evaluate differences in siring success between pollen from male and from hermaphrodite individuals.

In the present study, we compared the siring success (i.e. fruit set, fruit mass, seed number per fruit and mean seed mass) between male individuals that produce only staminate flowers and hermaphrodite individuals that produce only perfect flowers in the subdioecious shrub, *Eurya japonica*, in a field experiment using hand-pollination treatments. To explore the effects of the sex of the pollen recipient (female or hermaphrodite) on differences in siring success between male and hermaphrodite individuals, hand-pollination treatments were conducted with both female and hermaphrodite individuals as pollen recipients. The male fertility of male and hermaphrodite individuals was also assessed via progeny fitness, i.e. seed germination rate, germination timing and seedling survival rate in laboratory and greenhouse experiments using seeds produced by female individuals in the hand-pollination treatments.

## Methods

### Study species

*E. japonica* is a subdioecious evergreen broad-leaved shrub belonging to the Pentaphylacaceae; other subdioecious species also occur in *Eurya* (Wang *et al.* 2014). This species often grows in the lower layer of secondary and temperate evergreen broad-leaved forests in China, southern Korea and Japan (Ohwi and Kitagawa 1992; Oh *et al.* 1996). The flowers are pollinated by insects. They bloom between late February and early April, and the multi-seeded and bird-dispersed fruits ripen between late October and December. *E. japonica* has staminate, pistillate and perfect (i.e. hermaphrodite) flowers (Manabe *et al.* 1991; Murata *et al.* 1991; Higo 1994), and the pollen of perfect flowers is fertile (Manabe 1996). *E. japonica* individuals are classified into 6 sexual types: male individuals with only staminate flowers (M), female individuals with only pistillate flowers (F), hermaphrodite individuals with only perfect flowers (H), individuals with a mixture of perfect and staminate flowers (HM), individuals with a mixture of perfect and pistillate flowers (HF) and individuals with a mixture of all 3 flower types (HMF). In this study, we used male (M), female (F) and hermaphrodite (H) individuals that produce only pure-sexed flowers for the experiment because the intermediate sex phenotypes may affect the mating system in male and female functions. Sex change has been reported for 2 individuals (Tsuji and Sota 2013) in *E. japonica*, and was also observed frequently and repetitively in our population over 5 years (Wang *et al.* unpublished data).

### Study site and individuals

The study was conducted in three 20 × 20 m plots established close to each other (159.3–195.7 m) in February 2010 in a secondary forest at the Higashiyama Campus, Nagoya University, Japan (35°10′N, 136°58′E, 55, 80 m a.s.l.; for details of the study site, see Wang *et al.* 2015). Although the proportion of hermaphrodites including all sexual types in other populations is usually low (<1–10 %), but sometimes high (>10 %, reaching 38 %, Murata *et al.* 1991; Higo 1994; Manabe 1996; Suzuki 2005; Tsuji and Sota 2010), the sex ratio of *E. japonica* flowering individuals was M: 30.1 %; F: 33.2 %; H: 5.1 %; HF: 22.6 %; HM: 4.0 % and HFM: 2.4 % at our study site. To examine differences in siring success between pollen from male and from hermaphrodite individuals, 15 female and 9 hermaphrodite (Mother-H) individuals were used as mother trees (i.e. pollen recipients). In each plot, flowering individuals of 5 females and 2–5 hermaphrodites of approximately similar sizes (*P* = 0.23; diameter at ground level [D_0_]: 34.3 ± 11.2 and 37.8 ± 10.4 mm for female and hermaphrodite individuals, respectively; mean ± SD) and from similar light environments (*P* = 0.87; relative photosynthetic photon flux density: 12.2 ± 10.1 % and 11.4 ± 7.0 % for female and hermaphrodite individuals, respectively) were selected to limit the effects of resource availability in mother trees on siring success.

### Hand-pollination treatments

The pollen donors were 9 male (3 individuals per plot) and 9 hermaphrodite (Father-H, which were all also used as Mother-H) individuals. Due to the lack of sufficient hermaphrodite individuals to act as pollen recipients and pollen donors within each plot, pollen from male donors was collected exclusively from flowers of 3 different male individuals in the same plot for each mother individual, whereas hermaphrodite donors were collected from 3 different hermaphrodite individuals from any plot. No hermaphrodite individual was its own pollen donor, as hermaphrodites of *E. japonica* at the site were not self-compatible. For the hand-pollination treatments, a total of 12 shoots (about 20–25 cm in length) were randomly selected on each mother individual. All target shoots were tagged, and the number of flowers on each shoot (about 17 ± 8 flowers per shoot) was counted at peak flowering in late February 2011. These 12 target shoots per mother tree were randomly assigned to hand-pollination treatments (2 shoots for each of 3 male and 3 hermaphrodite donors). Regardless of the number of anthers per flower, to ensure that we could obtain sufficient pollen, we sampled enough staminate or perfect flowers, which were kept and thoroughly mixed in a plastic container. We then applied pollen to the stigmas of all flowers on target shoots using an artist’s paintbrush to ensure that the stigmas were completely coated in pollen. We brushed the stigmas gently 3 times using a brush with an adequate amount of pollen, and pollen appeared to be deposited evenly over stigmas of each flower. The anthers of each flower were naturally dehisced during hand-pollination. To avoid accidental mixing of pollen among pollen donors, we conducted hand-pollinations with careful attention, using different paintbrushes for each pollen donor. A total of 5618 flowers were hand-pollinated. Each shoot was bagged in a nylon mesh bag to exclude all potential pollinator visits before anthesis; the bags were removed for hand-pollination, and shoots were then rebagged.

Finally, we randomly selected 18–25 males and 3–4 hermaphrodites per plot including all the pollen donor individuals (a total of 69 males and 11 hermaphrodites, respectively), and measured the size (diameter at ground level [D_0_]) of each individual. Then, we counted the number of flowers per individual of all the pollen donor individuals. Additionally, as a preliminary experiment, the HF individuals were more abundant than hermaphrodites, 5–7 individuals per plot (18 individuals in total) and 6–8 shoots (about 20–25 cm in length; 114 shoots in total) per individual were randomly selected at each plot. We also counted the number of flowers per HF individual, and the number of pistillate and perfect flowers per shoot.

### Siring success

After the flowering season, the fate of flowers on each shoot was monitored until December 2011, when the fruit had matured. Mature fruits were collected and dried for 48 h at 80 °C, and the number of seeds per fruit was counted. Fruit mass was estimated as dry weight measured on 1–3 fruits per shoot. As the seeds of *E. japonica* were too small to measure individually, mean seed mass was calculated by dividing the total dry seed weight by the corresponding seed number. The remaining mature fruits from each female mother tree were not dried so that they could be used subsequently in the experiment to measure seed germination and seedling survival.

### Seed germination and seedling survival rates

To explore seed germination and seeding survival, we used 30–100 mature fruits from each female individual, as these individuals had sufficient fruit production for the seed germination and seedling experiments because of the greater female reproductive success of females relative to hermaphrodite individuals. The sampled fruits were stored for 2 months in a refrigerator at 4 °C. In early February 2012, after the pulp was removed, seeds were soaked in water for 1–2 days before sowing. We used 2302 seeds for the seed germination test (2 pollen donor sexes × 3 individual pollen donors × 3–30 seeds per pollen donor × 5 female individuals × 3 plots). The seeds per pollen donor were divided randomly into 2 replicates and sown on 90 × 15-mm Petri dishes. Each Petri dish contained 23–105 seeds (mean, 77 seeds) sired from 3 male or hermaphrodite pollen donor individuals, on one female individual. A total of 30 dishes were kept in a growth chamber under conditions of 14/10 h light and 24 °C/10 °C day/night for 91 days. These conditions were similar to natural conditions in May when many seeds germinate vigorously in the field. Germinated seeds were counted once every 2–5 days, and the filter paper in the dishes was kept suitably moist throughout the germination test.

For seedling survival, we planted 256 seeds (2 pollen donor sexes × 3 pollen donors × 1–3 seeds per pollen donor × 5 female individuals × 3 plots) that were treated in the same manner as in the seed germination test. Each pot contained seeds from 1 female individual, and 2 pollen donor sexes on the left and right sides. Each side of each pot included 1–3 seeds per pollen donor sired from 3 male and hermaphrodite pollen donor individuals, respectively. We placed seeds onto the surface of bog moss in humus-filled plastic pots (9.5 cm in depth, 11 cm in diameter). In early February 2012, 45 pots (3 replicates × 5 female individuals × 3 plots) were placed in a greenhouse. The fate of each seed was observed at intervals of a few weeks, and the survival of the 223 germinated seedlings was monitored until October 2012.

### Data analysis

To evaluate differences in siring success in terms of fruit set, fruit mass, seed number per fruit and mean seed mass between male and hermaphrodite individuals, we analysed the data using generalised linear mixed-effect models (GLMMs). In addition to the pollen donor sex (male vs. Father-H), the sex of the pollen recipient (mother sex; female vs. Mother-H) and their interactions were examined as fixed effects in the GLMMs. Mother individual, father individual and plot were set as random effects. To examine the effects of pollen donor sex on seed germination rate, seed germination day and seedling survival rate, we analysed the final fate (germinated or not) of each sown seed, the number of days until 50 % of seeds had germinated in each dish and the survival of the germinated seedlings as response variables in GLMMs. In the models, pollen donor sex was set as a fixed effect, and mother individual, father individual and plot were set as random effects. The statistical significance of the fixed effects was assessed using the *F*-test for Gaussian error distributions and *χ^2^*-test for Poisson and binomial error distributions. Post hoc comparisons were conducted by adjusting the family wise error based on Tukey’s method at *P* = 0.05. Data for fruit mass and mean seed mass were log-transformed before statistical analyses. Statistical analyses were performed using the R 3.0.1 software (R Development Core Team, 2013; *car*, *nlme*, *lme4*, *Mass* and *multcomp packages*). Response variables, number of samples, measurement units, random effects, error distributions and link functions for each analysis are summarised in Table 1.

**Table 1.** Summary of response variables, sample sizes, measurement units, random effects, error distributions and link functions used in the GLMM analyses. The data of fruit mass and mean seed mass were log-transformed before statistical analyses.

Response variables	No. of samples		Measurement units	Random effects	Error distributions	Link functions
	Male	Father-H				
Fruit set	142	143	Shoot	Plot, mother individual, father individual	Binomial	Logit
Fruit mass	356	299	Fruit	Plot, mother individual, father individual	Gaussian	Identity
Seed number	356	299	Fruit	Plot, mother individual, father individual	Poisson	Log
Mean seed mass	142	143	Shoot	Plot, mother individual, father individual	Gaussian	Identity
Seed germination rate	1269	1033	Seed	Plot, mother individual, father individual	Binomial	Logit
Seed germination day	30	30	Dish	Plot, mother individual, father individual	Poisson	Log
Seedling survival rate	117	106	Seedling	Plot, mother individual, father individual	Binomial	Logit

## Results

### Fruit production

**Figure 1:**
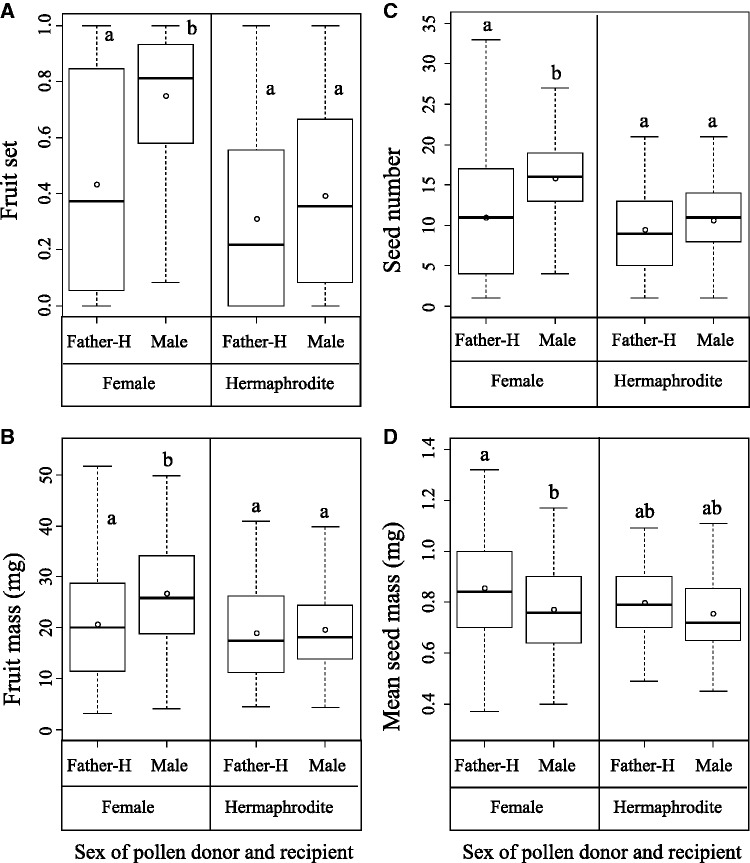
Box plots of siring success; (A) fruit set, (B) fruit mass, (C) seed numbers per fruit and (D) mean seed mass in female individuals and hermaphrodite individuals, which were hand-pollinated using pollen from hermaphrodites (Father-H) and males. The upper and lower hinges of the box indicate 75th and 25th percentiles of the data, respectively. The line and circle in the box show the median and mean of the data, respectively. Different letters indicate significant differences in tests of multiple comparisons, in which family wise errors were adjusted using Tukey’s method at *P* = 0.05.

The sex of both pollen donor and pollen recipient and the interaction between the two had significant effects on fruit set in *E. japonica* (Table 2). In female individuals, fruit set was significantly greater when flowers were hand-pollinated with pollen from male individuals than with that from hermaphrodite individuals, whereas no significant difference was found in hermaphrodite recipients in post hoc comparisons (Fig. 1A). A similar pattern was detected for fruit mass, although the effect of mother sex was marginally significant (Table 2). Fruits that were sired by male pollen were significantly heavier than those by hermaphrodite pollen in female individuals, but not in hermaphrodite individuals (Fig. 1B).

**Table 2.** Effects of pollen donor sex, mother sex, hand-pollination treatment and their interactions on fruit set, fruit mass, seed number per fruit and mean seed mass. To test the statistical significance of explanatory variables, the changes in deviance when each variable was removed from the full model were compared with *F*- or χ^2^ -distributions for Gaussian distributions or other distributions, respectively. Boldface indicates statistical significance.

Response variables	Fruit set			Fruit mass			Seed number			Seed mass		
Explanatory variables	df	χ^2^	*P*	df	*F*	*P*	df	χ^2^	*P*	df	*F*	*P*
Pollen donor sex (male vs.Father-H)	1	8.49	**<0.01**	1	13.33	**<0.001**	1	7.87	**<0.01**	1	12.13	**<0.001**
Mother sex (female vs. Mother-H)	1	8.19	**<0.01**	1	2.89	0.089	1	9.07	**<0.01**	1	1.57	0.210
Pollen donor sex × mother sex	1	18.14	**<0.001**	1	5.83	**<0.05**	1	8.19	**<0.01**	1	1.26	0.262

**Figure 2:**
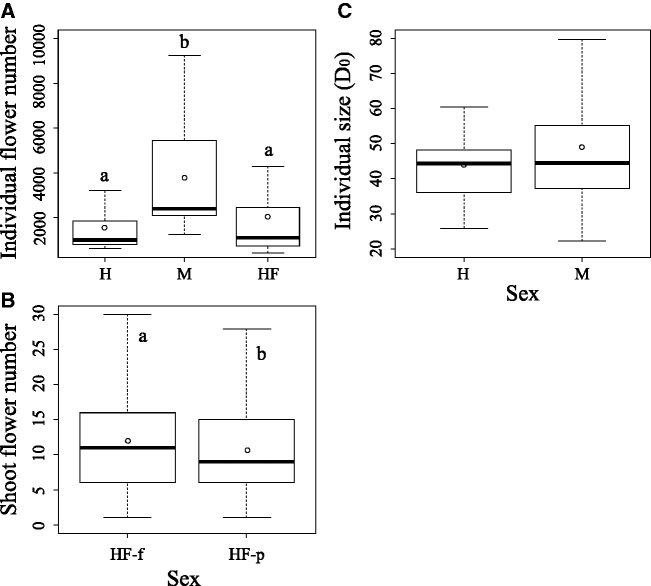
Box plots of (A) flower number per individual, (B) flower number per shoot and (C) size per individual of pollen donors between hermaphrodites (H) and males (M), and individuals with a mixture of pistillate and perfect flowers (HF). HF-f: pistillate flower of HF individual; HF-p: perfect flower of HF individual. The upper and lower hinges of the box indicate 75th and 25th percentiles of the data, respectively. The line and circle in the box show the median and mean of the data, respectively. Different letters indicate significant differences based on a generalised linear mixed model.

At the study site, male pollen donor individuals produced more flowers per individual than those of hermaphrodite pollen donor individuals (*P* < 0.001; Fig. 2A). In addition, the number of flowers per individual does not differ between HF individuals and hermaphrodite (*P* = 0.73), and HF individuals produced more pistillate than perfect flowers per shoot (*P* < 0.01; Fig. 2B). On the other hand, no difference was found in the size of both male and hermaphrodite individuals including all the pollen donors (*P* = 0.56; Fig. 2C).

### Seed production

Seed number per fruit was significantly affected by the sex of both pollen donor and pollen recipient and by the interaction (Table 2). When the pollen recipient was a female individual, fruits sired with the pollen of male individuals included more seeds than those sired with the pollen of hermaphrodite individuals, whereas there was no significant difference when the pollen recipient was a hermaphrodite individual (Fig. 1C). Only the sex of the pollen donor had a significant effect on mean seed mass (Table 2), and seeds sired with the pollen of hermaphrodite individuals were heavier than those sired with the pollen of males (Fig. 1D).

### Seed germination and seedling survival rates

**Figure 3:**
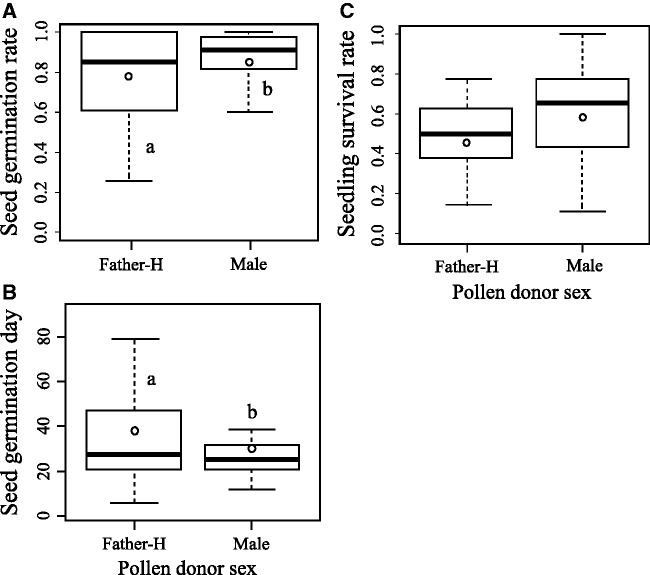
Box plots of (A) seed germination rate, (B) seed germination day and (C) seedling survival rate of seeds sired by single pollen of hermaphrodites (Father-H) and males (Male) in female individuals. Seed germination day was calculated for every Petri dish. The upper and lower hinges of the box indicate 75th and 25th percentiles of the data, respectively. The line and circle in the box show the median and mean of the data, respectively. Different letters indicate significant differences based on a generalised linear mixed model.

The sex of the pollen donor significantly affected the seed germination rate (*χ^2 ^*=^* *^16.9, df = 1, *P* < 0.001; Fig. 3A); seeds sired with the pollen of male individuals had a higher mean germination rate than those sired with the pollen of hermaphrodite individuals (86 % for male pollen vs. 78 % for hermaphrodite pollen). Seeds began to germinate 13 days after sowing and gradually germinated until 79 days after sowing. Average seed germination days were days 31 and 38 for seeds sired with the pollen of male and hermaphrodite individuals, respectively; thus, the seeds sired with hermaphrodite pollen took longer to germinate (*χ^2 ^*=^* *^34.9, df = 1, *P* < 0.001; Fig. 3B). Average seedling survival rates were 59 % and 46 % for seeds sired with the pollen of male and hermaphrodite individuals, respectively, but this difference was not significant (*χ^2 ^*=^* *^1.9, df = 1, *P* = 0.166; Fig. 3C).

## Discussion

### Higher siring success in males versus hermaphrodites

We observed a male advantage over hermaphrodites in male fertility by comparing siring success in a field experiment using hand-pollination treatment in the subdioecious *E. japonica*. This result was consistent with theoretical predictions (Charlesworth and Charlesworth 1978) and one empirical report for a subdioecious species (Morand-Prieyr *et al*. 2003). Male individuals outperformed hermaphrodites in terms of fruit set, fruit mass and seed number; overall, the pollen of male individuals sired more fruits and seeds than did that of hermaphrodites in *E. japonica*.

Male superiority occurred at the shoot level, and larger individuals and/or individuals that produced more flowers could potentially contribute to fruit and seed production more intensely at the individual level (Ishida and Hiura 1998; Philipp *et al.* 2009). At the study site, male individuals produced more flowers per individual than did hermaphrodites, although these individuals did not differ in size, suggesting that male individuals can potentially sire more fruits and seeds than can hermaphrodite individuals in *E. japonica*. Although HF individuals were more abundant than H individuals at the study site, the facts that the number of flowers per individual does not differ between HF and H individuals and the observation that HF individuals produced more pistillate than perfect flowers per shoot indicated that the male function of HF individuals is lower than that of H individuals. Therefore, pollen from male individuals should sire more fruits and seeds than pollen from all sexual types of hermaphrodite individuals (i.e. H, HF, HM and HFM) in the studied *E. japonica* population. On the other hand, when the pollen recipient was a female individual, seeds sired with the pollen of hermaphrodites were found to be heavier than those sired by pollen from males. This may have been caused by the trade-off between number and weight of seeds; the more seeds that are produced, the smaller the size of each seed is.

The relative fertility of male and hermaphrodite individuals (i.e. the M:H fertility ratio) in fruit and seed production (fruit set and seed number) of *E. japonica* ranged from 1.09 to 1.73 (mean: 1.41), which was greater than the result for subdioecious *S. acaulis* (0.94), but smaller than that of *F. excelsior* (≥10). In addition, the M:H fertility ratios were 1.57–1.73 and 1.09–1.26 when the mother sex was female and hermaphrodite, respectively. This prominent male advantage in females probably reflects higher female reproductive success in females over hermaphrodites rather than differences in receptivity of female and hermaphrodite individuals to pollen from male and hermaphrodite individuals.

### Progeny sired by male pollen had better quality

The higher germination rate of seeds on females sired by male pollen, despite their small size, indicated that male pollen can produce seeds of better quality compared with hermaphrodite pollen in *E. japonica*. This advantage of male pollen compared with hermaphrodite pollen was also observed in terms of seed germination day. Rapid seed germination may be profitable for acquiring ample space and plentiful photosynthetic products for seedling survival on the forest floor, where competition for resources among germinated plants is intense (Jones *et al.* 1997; Royo and Carson 2008; Rojas-Robles *et al.* 2012).

This result could have been due to the difference in amount of pollen applied on stigma because of the greater production of pollen in staminate than in perfect flowers (Wang *et al.* unpublished data). Previous studies indicated that the progeny from large pollen loads outperform progeny from small pollen loads in terms of germination rate and growth rate (Winsor *et al.* 1987; Snow 1991; Quesada *et al.* 1993). The difference in the size of the pollen load deposited on stigmas often causes a difference in the number of seeds per fruit (Snow 1982; Shore and Barrett 1984; Winsor *et al.* 1987). It is also able to reduce fruit set because fruits with fewer fertilized seeds are less likely to mature. However, although we did not count the number of pollen grains, we brushed the stigmas with an adequate amount of pollen to ensure saturation with pollen for both male and hermaphrodite pollen. Therefore, the male advantage in male fertility was likely caused mainly by the better quality of male pollen. The similar size of pollen load could explain the better quality of progeny due to differences in pollen quality, such as higher pollen germination rate and faster growing pollen tubes. Several studies showed that seeds produced by the fastest pollen tubes germinate more quickly and grow faster than those produced by slower tubes (Mulcahy 1983; Winsor *et al.* 1987; Quesada *et al.* 1993). Further studies on the pollen quality of male and hermaphrodite individuals are needed to explore the precise mechanisms underlying the differences in progeny performance.

Although no differences in seedling survival rate were observed between *E. japonica* seedlings sired with the pollen of male and those sired by application of hermaphrodite pollen under standardised resource-rich conditions (i.e. greenhouse), differences in seedling performance may emerge in the field (i.e. forest floor), where conditions are more severe. The seedlings sired with male pollen of higher quality are expected to show better survival. Further field survey data as well as genetic experiments are necessary to understand and quantify actual seedling performance in *E. japonica*.

When we calculated the relative fertility among females, males and hermaphrodites using the values of fruit set × seed number in hand-pollinated crosses, the relative fertility of H_total_, which was the total relative fertility of all sexual types of hermaphrodites (H, HF, HM, HFM) via female and male functions, was smaller than those of females and males (F:M:H_total_ = 1:1:0.61). Spigler and Ashman (2012) have pointed out the importance of pollen limitation as a factor influencing the late stage of the pathway to dioecy via gynodioecy. Previous work showed that trioecy (subdiocy) can be maintained under pollen limitation of female seed production because pollen limitation reduces the fitness of females but not self-fertile hermaphrodites, counteracting the seed fertility advantage of females in subdioecious population (Maurice and Fleming 1995; Fleming *et al.* 1998; Wolf and Takebayashi 2004). However, in self-incompatible *E. japonica*, considering the weakened reproductive advantage of females against hermaphrodites under natural conditions (Wang *et al.* 2015), although *E. japonica* is transitioning toward dioecy, pollinator-mediated pollen limitation may suppress enhanced fertility of females and males, thereby promoting the persistence of hermaphrodites in this *E. japonica* population (i.e. stable subdioecy).

## Conclusions

Male individuals exhibited an advantage in male fertility compared with hermaphrodite individuals in hand-pollinated crosses in subdioecious species *E. japonica*. This male advantage was prominent when the mother trees were female individuals and not hermaphrodites. Hand-pollination treatments also revealed that seeds sired with pollen from male individuals exhibited better performance. Given that the female reproductive success of female individuals is higher than that of hermaphrodites and that hermaphrodites are self-incompatible in *E. japonica* (Wang *et al.* 2015), pollen limitation may inhibit the shift and permit the persistence of hermaphrodites in this *E. japonica* population.

## Sources of Funding

Our work was funded by University expense of Nagoya University, National Natural Science Foundation of China (Youth Fund Project) (31600313), Science and Technology Project of Shandong Province (No. 2014GZX217005) and China Postdoctoral Science Foundation (2016M592176).

## Contributions by the Authors

H.W., M.M. and M.N. conceptualized and designed the research; H.W. conducted field work; H.W., M.M. and M.N. analyzed the data; H.W., N.T. and M.N. wrote the manuscript.

## Conflict of Interest Statement

None declared.
